# A bug’s life: Delving into the challenges of helminth microbiome studies

**DOI:** 10.1371/journal.pntd.0008446

**Published:** 2020-09-10

**Authors:** Fabio Formenti, Alba Cortés, Paul J. Brindley, Cinzia Cantacessi, Gabriel Rinaldi

**Affiliations:** 1 Department of Veterinary Medicine, University of Cambridge, Cambridge, United Kingdom; 2 IRCCS Sacro Cuore Don Calabria Hospital, Negrar, Verona, Italy; 3 Departament de Farmàcia i Tecnologia Farmacèutica i Parasitologia, Facultat de Farmàcia, Universitat de València, Burjassot, València, Spain; 4 Department of Microbiology, Immunology and Tropical Medicine, and Research Center for Neglected Diseases of Poverty, School of Medicine & Health Sciences, George Washington University, Washington, DC, United States of America; 5 Wellcome Sanger Institute, Wellcome Genome Campus, Hinxton, United Kingdom; University of Calgary, CANADA

The body of vertebrates is inhabited by trillions of microorganisms, i.e., viruses, archaea, bacteria, and unicellular eukaryotes, together referred to as the “microbiota.” Similarly, vertebrates also host a plethora of parasitic worms (the “macrobiota”), some of which share their environment with the microbiota inhabiting the gastrointestinal (GI) tract [1]. Complex interactions between the helminths and the gut microbiota have been associated with establishment of parasite infection, disease manifestations, and host immune-modulation [2, 3]. Remarkably, not only GI helminths alter the gut microbiome composition [4], but also the infections with blood flukes of the genus *Schistosoma* have been associated with intestinal dysbiosis, that even occurs before the onset of egg laying [5, 6]. Comparably, over the last decade, evidence has emerged of the contribution(s) of the resident microbiota to several physiological and reproductive processes of invertebrate hosts, including insects, arachnids, worms, and snails [7, 8]. These noteworthy discoveries, coupled with the recent expansion of high-throughput microbiota- and microbiome-profiling approaches (the former referring to the community of microorganisms themselves and the latter to the microorganisms and their genomes, within a given ecological niche), are rapidly leading to a much better understanding of the composition and functions of microbial communities inhabiting parasitic worms of major public health and socioeconomic significance. This basic knowledge might expose exploitable vulnerabilities of parasites, thus paving the way to the development of novel control strategies [9].

In this Viewpoint, we consider the challenges associated with the study of the helminth microbiota/-me, spanning not only bacteria transiently associated with parasites in which the life cycle includes free-living and parasitic stages, but also putative helminth endosymbionts. Indeed, endosymbionts have been described in both roundworms and flatworms [10, 11]. In nematodes, the most notable example of a mutualistic relationship between worms and bacteria is represented by filarial parasites [12], i.e., *Onchocerca volvulus*, *Wuchereria bancrofti*, and *Brugia malayi*, agents of human lymphatic filariasis. In particular, the fitness, propagation, and survival of these worms depend on endosymbiotic bacteria of the genus *Wolbachia* that have thus become the target of intense research aimed to develop novel filaricidal compounds [11, 13]. On the other hand, bacteria of the genus *Neorickettsia* have been identified in the endoparasitic digeneans, i.e., trematodes [10]. These intracellular bacteria inhabit the worm reproductive tissues and are vertically-transmitted to the next generation of parasites *via* the eggs [10]. In addition, horizontal transmission of *Neorickettsia* from the fluke to the fluke-infected vertebrate host, where the bacteria colonize macrophages among other cell types, is a determinant for the pathogenesis of severe disease in, for example, horses, dogs, and humans [14]. Recently, we have sequenced and characterized the whole genome of a *Neorickettsia* endobacterium in an isolate of adult *Fasciola hepatica* liver flukes [15]. The *Neorickettsia*, related to the etiological agents of human Sennetsu and Potomac horse fevers, was localized in the gonads of the liver fluke and its DNA detected by PCR in eggs, thus supporting a germline transmission [15].

To decipher the role of parasite-associated microbiota on the pathophysiology of helminthiases, the Parasite Microbiome Project (PMP) was launched in January 2019 [9]. Importantly, the PMP encourages best practices for experimental designs to ensure robust and reliable comparisons between datasets and promotes the inclusion of appropriate controls to correctly identify environmental microbial contaminants [9]. These practices are particularly important in experiments in which microbiota profiling is conducted using next generation sequencing (NGS) (i.e., high-throughput) technologies that are particularly prone to exogenous bacterial contamination, such as bacterial 16S rRNA-amplicon sequencing on low-biomass samples, e.g., helminths [16, 17]. Therefore, given the potential confounders in helminth-associated microbiome studies, we propose that four elements, outlined below, must be considered in order to generate reliable and reproducible data (Fig 1).

**Fig 1:**
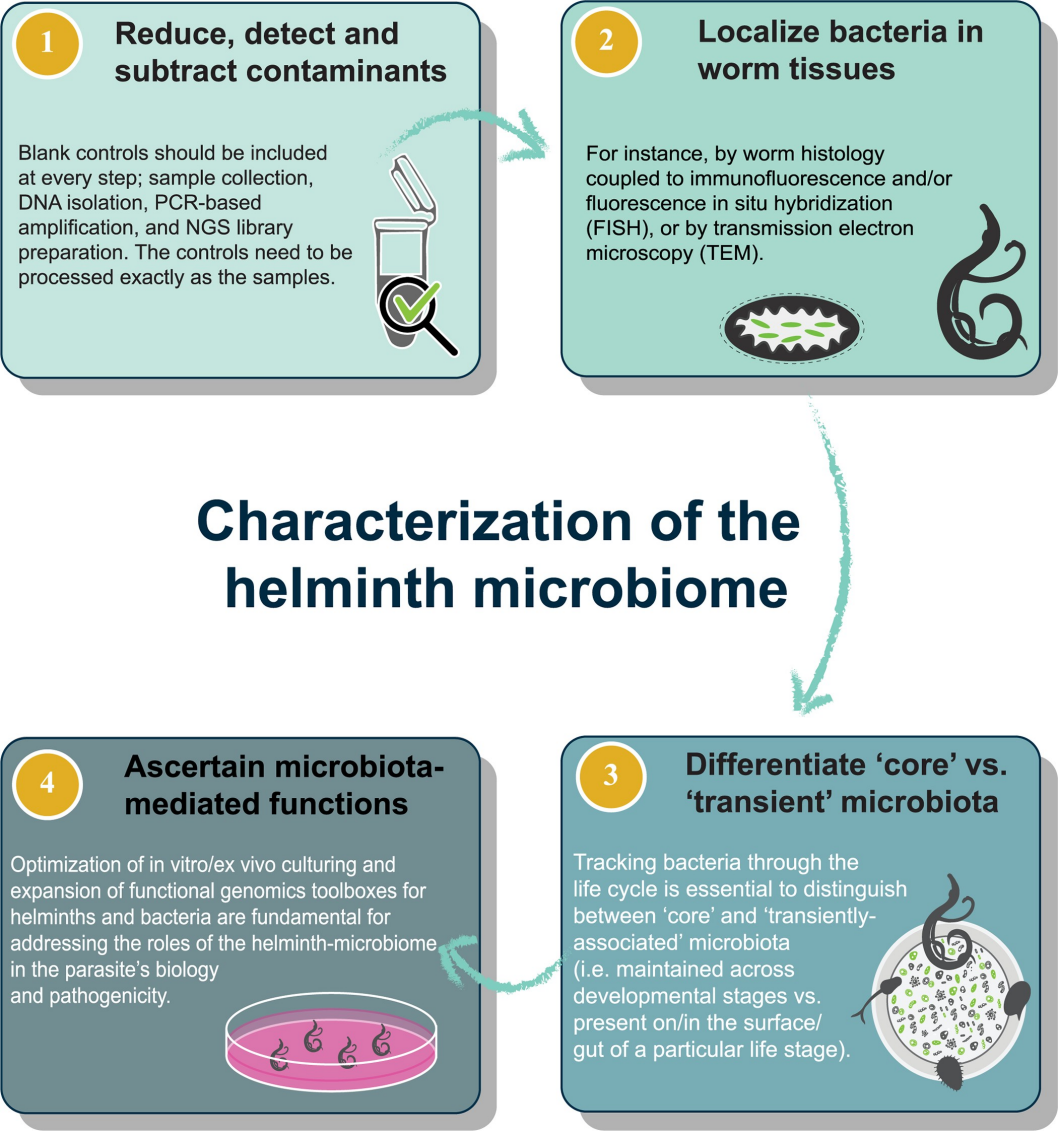
Key elements for a reliable and reproducible characterization of the helminth-associated microbiome. NGS; next generation sequencing.

## 1: Appropriate controls

The identification and characterization of the helminth-associated microbiota/-me includes several experimental steps from sample collection to library preparation and sequencing, each of which is exposed to different sources of contamination. Therefore, the inclusion of matching negative controls (“blanks”) in each step of the experiment is critical. The sample collection should ideally be carried out under clean conditions by using disposable sterile consumables and autoclaved instruments to minimize the risk of sample contamination with environmental bacteria. In addition, controls for each tentative source of environmental contaminants should be included. Following thorough screening, the sequence data generated from these negative controls from each experimental step can be subtracted from the datasets under consideration.

## 2: Microscopical visualisation of helminth-associated bacteria

Following *in silico* subtraction of putative contaminant sequences, unequivocal identification and characterization of worm microbiomes can rely on microscopical techniques aimed to localize bacteria of interest across different helminth tissues and developmental stages. Widely used approaches to localize specific groups of microorganisms are based on fluorochromes conjugated to either antibodies or nuclei acid probes that bind to specific bacterial proteins or nuclei acids, respectively. *Neorickettsia* bacteria were identified within the reproductive tissue of the liver fluke *F*. *hepatica via* fluorescent immunohistochemistry [15], whereas a recent report characterized the “core microbiome” associated with the ovine GI nematode *Haemonchus contortus* using fluorescence *in situ* hybridization and light and transmission electron microscopy [18].

## 3: “Core microbiome” *versus* transiently associated bacteria

The localization of microorganisms in helminth tissues is a robust indication of the occurrence of a worm microbiome, and may provide clues on its function(s); for instance, *Wolbachia* localized in the reproductive tissues of filarial parasites have been shown to be involved in sexual differentiation [19] and worm survival [20]. However, the distinction between bacteria that might be transiently associated with the parasite, e.g., coating the surface of free-living larval stages or transported within the alimentary tract of the parasite among different host niches, and those that might belong to the worm “core microbiome” is crucial.

Protocols to eliminate bacteria contaminating the external tegument of GI worms have been implemented. Treatment of *Trichuris muris* worms with sodium hypochlorite allowed the identification of a specific parasite intestinal microbiota distinct from that of its host [2]. Whether transiently associated bacteria have direct effects on parasite biology still needs to be ascertained; notwithstanding, they might contribute to the pathophysiology of the infection and comorbidity in the host. The Asian liver fluke *Opisthorchis viverrini* provides a signal example in this regard; accumulating evidence suggests that the juvenile form of the parasite excysted in the duodenum of the human host might ingest *Helicobacter pylori* and/or related species of bacteria and transport the bacillus to the bile duct, where this fluke establishes [21, 22]. Chronic infection with either *H*. *pylori* bacteria or *O*. *viverrini* is classified by the International Agency for Research on Cancer as a Group 1 carcinogen, leading to gastric adenocarcinoma or cholangiocarcinoma (CCA), respectively [23]. We have previously reported a synergistic association between the liver fluke and *Helicobacter* bacteria in the development of the opisthorchiasis-associated CCA [21], which may result from an eventual horizontal transmission of bacteria from the parasite to host tissue. On the other hand, a worm “core microbiome” (particularly, if associated with the parasite gonads) may be vertically-transmitted to the next generation and, hence, detected across different developmental stages. Therefore, screening for the presence of bacteria in different developmental stages of the parasite, either by PCR, qPCR, and/or 16S-rRNA amplicon NGS [15, 18] is recommended to define the “core microbiome” that might serve as a foundation to explore novel strategies for transmission control. In addition to bacteria, the “core microbiome” may comprise microeukaryotes, such as fungi and protozoa, and viruses that can be detected by shotgun metagenomic approaches [9]. Although these methods are not inexpensive and generate complex data (which require advanced bioinformatics analyses that include the identification and *in silico* subtraction of helminth-derived sequences), their application is recommended to gain an overall picture of the helminth microbiome and enable the prediction of bacterial metabolic pathways that might be essential for worm biology and (patho)physiology associated with the infection [24]. Subsequently, the use of functional approaches to investigate the roles of this “core microbiome” in worm biology, helminth infection, establishment, and host–parasite interactions becomes critical.

## 4: Functional studies of the helminth-associated microbiome

The follow-on step after the identification of both transiently associated bacteria and the worm “core microbiome” is to understand the biological relevance of these interactions. The use of broad- or narrow-spectrum antibiotics to alter the worm microbiome might assist the determination of the essentiality of these bacteria for worm survival, fitness, and/or reproduction [25]. In addition, optimization of protocols for in vitro and *ex vivo* culturing of parasitic developmental stages [26–28], and the use of organoids to simulate interactions between parasites, host cells, and selected bacteria [29], in tandem with functional genomic tools currently under development for helminths (e.g., genome editing [30–32]) and bacteria [33] will assist the set-up of controlled experiments to address hypotheses on mechanisms underlying worm-microbes interactions. Similar approaches have been employed for model organisms such as *Caenorhabditis elegans*. The *C*. *elegans*–associated microbiome has been analyzed in laboratory settings by culturing worms on individual bacterial strains and evaluating helminth growth rate and responses of stress and immune related genes. The majority of the bacterial strains investigated were found to be beneficial for worm fitness [34]. Finally, where feasible, *in vivo* studies using rodent models of helminth infection might provide invaluable functional insights on transmission of bacteria across parasitic developmental stages, microbial horizontal translocation to host tissues, and bacteria-mediated pathologies associated with helminthiases. Germ-free and gnotobiotic mice (i.e., animals exclusively colonised by known microbes) are extensively used in microbiome studies [35]. However, the dysfunctional immune response of these animals might add several confounders to the infection model. On the other hand, the use of antibiotics in well-established murine models of helminthiases might allow to target specific groups of host- and/or worm-associated bacteria.

To conclude, similarly to the human microbiome, bacteria associated with helminth parasites likely represent an intrinsic part of these organisms, so much so that parasite biology might not be completely understood in its absence. However, the characterization of the genuine helminth microbiome may turn out to be complex, or even daunting, due to several technical challenges. In our view, rigorous hygiene to exclude or at least minimize contaminants, together with bacterial localization in helminth tissues and across developmental stages, and their functional characterization are essential steps to unequivocally identify bacteria associated with parasitic worms and ascertain their roles in the dynamic crosstalk among the parasite, the host, and the host microbiome. Ultimately, this will contribute to the current incomplete understanding of the biology and pathogenicity of helminths.

## References

[1] Cortés A, Peachey L, Scotti R, Jenkins TP, Cantacessi C. Helminth-microbiota cross-talk—A journey through the vertebrate digestive system. Mol Biochem Parasitol. 2019;233:111222 Epub 2019/09/22. 10.1016/j.molbiopara.2019.111222 . 31541662

[2] White EC, Houlden A, Bancroft AJ, Hayes KS, Goldrick M, Grencis RK, et al Manipulation of host and parasite microbiotas: Survival strategies during chronic nematode infection. Sci Adv. 2018;4(3):eaap7399. Epub 2018/03/17. 10.1126/sciadv.aap7399 29546242 PMC5851687

[3] Holzscheiter M, Layland LE, Loffredo-Verde E, Mair K, Vogelmann R, Langer R, et al Lack of host gut microbiota alters immune responses and intestinal granuloma formation during schistosomiasis. Clin Exp Immunol. 2014;175(2):246–57. Epub 2013/10/31. 10.1111/cei.12230 24168057 PMC3892416

[4] Rapin A, Chuat A, Lebon L, Zaiss MM, Marsland BJ, Harris NL. Infection with a small intestinal helminth, *Heligmosomoides polygyrus bakeri*, consistently alters microbial communities throughout the murine small and large intestine. Int J Parasitol. 2020;50(1):35–46. Epub 2019/11/25. 10.1016/j.ijpara.2019.09.005 . 31759944

[5] Zhao Y, Yang S, Li B, Li W, Wang J, Chen Z, et al Alterations of the Mice Gut Microbiome via *Schistosoma japonicum* Ova-Induced Granuloma. Front Microbiol. 2019;10:352 Epub 2019/03/21. 10.3389/fmicb.2019.00352 30891012 PMC6411663

[6] Jenkins TP, Peachey LE, Ajami NJ, MacDonald AS, Hsieh MH, Brindley PJ, et al *Schistosoma mansoni* infection is associated with quantitative and qualitative modifications of the mammalian intestinal microbiota. Sci Rep. 2018;8(1):12072 Epub 2018/08/15. 10.1038/s41598-018-30412-x 30104612 PMC6089957

[7] Engel P, Moran NA. The gut microbiota of insects—diversity in structure and function. FEMS Microbiol Rev. 2013;37(5):699–735. Epub 2013/05/23. 10.1111/1574-6976.12025 . 23692388

[8] Huot C, Clerissi C, Gourbal B, Galinier R, Duval D, Toulza E. Schistosomiasis Vector Snails and Their Microbiota Display a Phylosymbiosis Pattern. Front Microbiol. 2019;10:3092 Epub 2020/02/23. 10.3389/fmicb.2019.03092 32082267 PMC7006369

[9] Dheilly NM, Martinez Martinez J, Rosario K, Brindley PJ, Fichorova RN, Kaye JZ, et al Parasite microbiome project: Grand challenges. PLoS Pathog. 2019;15(10):e1008028 Epub 2019/10/11. 10.1371/journal.ppat.1008028 31600339 PMC6786532

[10] Greiman SE, Tkach VV, Vaughan JA. Transmission rates of the bacterial endosymbiont, *Neorickettsia risticii*, during the asexual reproduction phase of its digenean host, *Plagiorchis elegans*, within naturally infected lymnaeid snails. Parasit Vectors. 2013;6:303 Epub 2014/01/05. 10.1186/1756-3305-6-303 24383453 PMC3924192

[11] Slatko BE, Taylor MJ, Foster JM. The *Wolbachia* endosymbiont as an anti-filarial nematode target. Symbiosis. 2010;51(1):55–65. Epub 2010/08/24. 10.1007/s13199-010-0067-1 20730111 PMC2918796

[12] Bouchery T, Lefoulon E, Karadjian G, Nieguitsila A, Martin C. The symbiotic role of *Wolbachia* in Onchocercidae and its impact on filariasis. Clin Microbiol Infect. 2013;19(2):131–40. Epub 2013/02/13. 10.1111/1469-0691.12069 . 23398406

[13] Johnston KL, Cook DAN, Berry NG, David Hong W, Clare RH, Goddard M, et al Identification and prioritization of novel anti-*Wolbachia* chemotypes from screening a 10,000-compound diversity library. Sci Adv. 2017;3(9):eaao1551 Epub 2017/09/30. 10.1126/sciadv.aao1551 28959730 PMC5617373

[14] Vaughan JA, Tkach VV, Greiman SE. Neorickettsial endosymbionts of the digenea: diversity, transmission and distribution. Adv Parasitol. 2012;79:253–97. Epub 2012/06/26. 10.1016/B978-0-12-398457-9.00003-2 . 22726644

[15] McNulty SN, Tort JF, Rinaldi G, Fischer K, Rosa BA, Smircich P, et al Genomes of *Fasciola hepatica* from the Americas Reveal Colonization with *Neorickettsia* Endobacteria Related to the Agents of Potomac Horse and Human Sennetsu Fevers. PLoS Genet. 2017;13(1):e1006537 Epub 2017/01/07. 10.1371/journal.pgen.1006537 28060841 PMC5257007

[16] Eisenhofer R, Minich JJ, Marotz C, Cooper A, Knight R, Weyrich LS. Contamination in Low Microbial Biomass Microbiome Studies: Issues and Recommendations. Trends Microbiol. 2019;27(2):105–17. Epub 2018/12/01. 10.1016/j.tim.2018.11.003 . 30497919

[17] Salter SJ, Cox MJ, Turek EM, Calus ST, Cookson WO, Moffatt MF, et al Reagent and laboratory contamination can critically impact sequence-based microbiome analyses. BMC Biol. 2014;12:87 Epub 2014/11/13. 10.1186/s12915-014-0087-z 25387460 PMC4228153

[18] Sinnathamby G, Henderson G, Umair S, Janssen P, Bland R, Simpson H. The bacterial community associated with the sheep gastrointestinal nematode parasite *Haemonchus contortus*. PLoS ONE. 2018;13(2):e0192164 Epub 2018/02/09. 10.1371/journal.pone.0192164 29420571 PMC5805237

[19] Negri I, Pellecchia M, Greve P, Daffonchio D, Bandi C, Alma A. Sex and stripping: The key to the intimate relationship between *Wolbachia* and host? Commun Integr Biol. 2010;3(2):110–5. Epub 2010/06/30. 10.4161/cib.3.2.10520 20585501 PMC2889965

[20] Casiraghi M, McCall JW, Simoncini L, Kramer LH, Sacchi L, Genchi C, et al Tetracycline treatment and sex-ratio distortion: a role for *Wolbachia* in the moulting of filarial nematodes? Int J Parasitol. 2002;32(12):1457–68. Epub 2002/10/24. 10.1016/s0020-7519(02)00158-3 . 12392911

[21] Deenonpoe R, Mairiang E, Mairiang P, Pairojkul C, Chamgramol Y, Rinaldi G, et al Elevated prevalence of *Helicobacter* species and virulence factors in opisthorchiasis and associated hepatobiliary disease. Sci Rep. 2017;7:42744 Epub 2017/02/16. 10.1038/srep42744 28198451 PMC5309894

[22] Sripa B, Deenonpoe R, Brindley PJ. Co-infections with liver fluke and *Helicobacter* species: A paradigm change in pathogenesis of opisthorchiasis and cholangiocarcinoma? Parasitol Int. 2017;66(4):383–9. Epub 2016/12/07. 10.1016/j.parint.2016.11.016 27919744 PMC5457716

[23] Humans IWGotEoCRt. Biological agents. Volume 100 B. A review of human carcinogens. IARC Monogr Eval Carcinog Risks Hum. 2012;100(Pt B):1–441. Epub 2012/11/30. 23189750 PMC4781184

[24] Zhang X, Li L, Butcher J, Stintzi A, Figeys D. Advancing functional and translational microbiome research using meta-omics approaches. Microbiome. 2019;7(1):154 Epub 2019/12/08. 10.1186/s40168-019-0767-6 31810497 PMC6898977

[25] Taylor MJ, Voronin D, Johnston KL, Ford L. *Wolbachia* filarial interactions. Cell Microbiol. 2013;15(4):520–6. Epub 2012/12/06. 10.1111/cmi.12084 . 23210448

[26] Mann VH, Morales ME, Rinaldi G, Brindley PJ. Culture for genetic manipulation of developmental stages of *Schistosoma mansoni*. Parasitology. 2010;137(3):451–62. Epub 2009/09/22. 10.1017/S0031182009991211 19765348 PMC3042131

[27] McCusker P, McVeigh P, Rathinasamy V, Toet H, McCammick E, O'Connor A, et al Stimulating Neoblast-Like Cell Proliferation in Juvenile *Fasciola hepatica* Supports Growth and Progression towards the Adult Phenotype *In Vitro*. PLoS Negl Trop Dis. 2016;10(9):e0004994 Epub 2016/09/14. 10.1371/journal.pntd.0004994 27622752 PMC5021332

[28] Wang J, Chen R, Collins JJ, 3rd. Systematically improved *in vitro* culture conditions reveal new insights into the reproductive biology of the human parasite *Schistosoma mansoni*. PLoS Biol. 2019;17(5):e3000254 Epub 2019/05/09. 10.1371/journal.pbio.3000254 31067225 PMC6505934

[29] Duque-Correa MA, Maizels RM, Grencis RK, Berriman M. Organoids—New Models for Host-Helminth Interactions. Trends Parasitol. 2020;36(2):170–81. Epub 2019/12/04. 10.1016/j.pt.2019.10.013 . 31791691 PMC7106373

[30] Arunsan P, Ittiprasert W, Smout MJ, Cochran CJ, Mann VH, Chaiyadet S, et al Programmed knockout mutation of liver fluke granulin attenuates virulence of infection-induced hepatobiliary morbidity. Elife. 2019;8 Epub 2019/01/16. 10.7554/eLife.41463 30644359 PMC6355195

[31] Gang SS, Castelletto ML, Bryant AS, Yang E, Mancuso N, Lopez JB, et al Targeted mutagenesis in a human-parasitic nematode. PLoS Pathog. 2017;13(10):e1006675 Epub 2017/10/11. 10.1371/journal.ppat.1006675 29016680 PMC5650185

[32] Ittiprasert W, Mann VH, Karinshak SE, Coghlan A, Rinaldi G, Sankaranarayanan G, et al Programmed genome editing of the omega-1 ribonuclease of the blood fluke, *Schistosoma mansoni*. Elife. 2019;8 Epub 2019/01/16. 10.7554/eLife.41337 30644357 PMC6355194

[33] Bikard D, Euler CW, Jiang W, Nussenzweig PM, Goldberg GW, Duportet X, et al Exploiting CRISPR-Cas nucleases to produce sequence-specific antimicrobials. Nat Biotechnol. 2014;32(11):1146–50. Epub 2014/10/06. 10.1038/nbt.3043 25282355 PMC4317352

[34] Samuel BS, Rowedder H, Braendle C, Felix MA, Ruvkun G. *Caenorhabditis elegans* responses to bacteria from its natural habitats. Proc Natl Acad Sci U S A. 2016;113(27):E3941–9. Epub 2016/06/19. 10.1073/pnas.1607183113 27317746 PMC4941482

[35] Kennedy EA, King KY, Baldridge MT. Mouse Microbiota Models: Comparing Germ-Free Mice and Antibiotics Treatment as Tools for Modifying Gut Bacteria. Front Physiol. 2018;9:1534 Epub 2018/11/16. 10.3389/fphys.2018.01534 30429801 PMC6220354

